# Evolution of dosage compensation under sexual selection differs between X and Z chromosomes

**DOI:** 10.1038/ncomms8720

**Published:** 2015-07-27

**Authors:** Charles Mullon, Alison E. Wright, Max Reuter, Andrew Pomiankowski, Judith E. Mank

**Affiliations:** 1 Department of Genetics, Evolution and Environment, University College London, Gower Street, London WC1E 6BT, UK; 2 CoMPLEX, University College London, Gower Street, London WC1E 6BT, UK; 3 Department of Ecology and Evolution, Biophore, University of Lausanne, Ch-1015 Lausanne, Switzerland; 4 Department of Zoology, University of Oxford, The Tinbergen Building, South Parks Road, Oxford OX1 3PS, UK

## Abstract

Complete sex chromosome dosage compensation has more often been observed in XY than ZW species. In this study, using a population genetic model and the chicken transcriptome, we assess whether sexual conflict can account for this difference. Sexual conflict over expression is inevitable when mutation effects are correlated across the sexes, as compensatory mutations in the heterogametic sex lead to hyperexpression in the homogametic sex. Coupled with stronger selection and greater reproductive variance in males, this results in slower and less complete evolution of Z compared with X dosage compensation. Using expression variance as a measure of selection strength, we find that, as predicted by the model, dosage compensation in the chicken is most pronounced in genes that are under strong selection biased towards females. Our study explains the pattern of weak dosage compensation in ZW systems, and suggests that sexual selection plays a major role in shaping sex chromosome dosage compensation.

## Abstract (web summary)

Complete sex chromosome dosage compensation is largely limited to male heterogametic species, with the majority of female heterogametic species displaying incomplete dosage compensation. Here, the authors show that sexual conflict over gene expression combined with sexual selection in males can explain this pattern.

## Graphical abstract

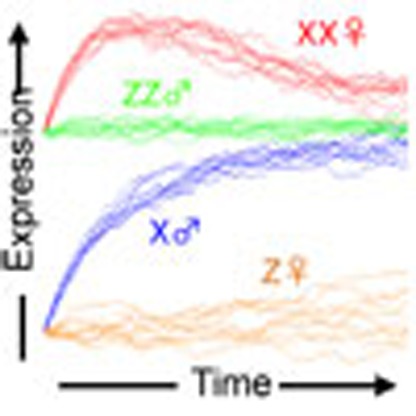

Sex chromosomes diverge following the suppression of recombination, which results in the decay of the non-recombining Y or W sex chromosomes^1^. The degradation of Y- and W-linked genes causes a reduction in expression levels in the heterogametic sex^2^,^3^, which retains only one copy of X- or Z-linked loci. This decrease in expression can have a negative effect on the fitness of the heterogametic sex (XY in male and ZW in female heterogamety). The deleterious effects of Y and W degradation are thought to drive the evolution of dosage compensation, where selection acts to increase expression in the heterogametic sex and so restore the expression level that existed prior to sex chromosome divergence^4^,^5^.

Ohno^4^ suggested that complete dosage compensation would follow gene loss on the Y or W chromosome, and many assumed that complete dosage compensation was an essential feature of all heteromorphic sex chromosomes. However, expression data shows that dosage compensation varies widely among taxa. Some species, such as *Drosophila melanogaster*^6^,^7^ and *Caenorhabditis elegans*^8^,^9^, achieve near complete dosage compensation across the entire X chromosome. This has been observed in several other male heterogametic (XY) species (Table 1). In contrast, complete dosage parity has only been observed in one female heterogametic (ZW) species^10^. All other female heterogametic species thus far studied display incomplete dosage compensation, with some individual genes compensated, but the average expression of female Z-linked genes below that of the two copies in males, and below the average expression of autosomal copies in females (Table 1, Supplementary Table 1). Age of sex chromosome system does not explain the distribution of incomplete versus complete dosage compensation, as both birds and Lepidoptera possess relatively old, highly conserved sex chromosome systems that are largely uncompensated^11^. The term incomplete dosage compensation in Table 1 includes several different possible regulatory states, ranging from dosage compensation for a subset of genes to partial compensation for a large proportion of genes. Although it is not possible to differentiate these various forms of incomplete dosage compensation, the striking difference between male and female heterogamety suggests that the evolution of dosage compensation is influenced by different forces in X and Z chromosomes.

Here we build a population genetic model in which dosage compensation arises through the accumulation of *cis*-regulatory mutations that alter the expression of X- or Z-linked genes^12^. To be consistent with our subsequent data analysis, we define dosage compensation as the evolution of equal expression in males and females following the loss of the Y or W allele in the heterogametic sex. As correlations in expression between the sexes are typically strongly positive^13^,^14^, we allow mutations altering expression in one sex to cause correlated change in the other sex. Therefore, mutations that increase expression in the heterogametic sex to compensate for reduced unbalanced selection between the sexes may lead to hyperexpression in the homogametic sex, resulting in sexually antagonistic fitness effects. When there is sexual conflict, the adaptive response is dominated by the sex under stronger selection^15^,^16^. In addition, in the case of sex chromosomes, selection is greater in the homogametic sex relative to that in the heterogametic sex^17^. As the Z chromosome resides two-thirds of the time in males while the X chromosomes resides two-thirds of the time in females, selection on males has a disproportionate effect on the Z and selection on females has a disproportionate effect on the X. Finally, the efficacy of selection on a mutation can differ according to its location on the Z or the X chromosome. Reproductive variance may vary between the sexes, and is often higher in males^18^,^19^. This affects Z and X chromosomes differently, as the Z chromosome is more sensitive to increased male reproductive variance than the X^16^,^20^,^21^,^22^, lowering the rate of adaptation on the Z relative to that on the X. We examine how the combination of these forces results in differences between XY and ZW systems in the evolutionary rates of dosage compensation, and find that slower and less complete Z dosage compensation evolution is associated with a positive correlation in mutational effects across the sexes, stronger selection on males and greater male reproductive variance. Our model also predicts that dosage compensation should be most pronounced in genes that are under strong selection, biased towards the heterogametic sex. Using expression variance as a measure of selection strength on expression, we find support for these predictions from the chicken transcriptome.

## Results

### Rates of dosage compensation evolution

In the model (see Methods for details), expression evolves via the recurrent fixation of *cis*-regulatory mutations of small effect, consistent with previous studies of gene expression evolution^23^. We assume that mutations are sufficiently rare for each one to be fixed or lost before another arises. Mutations cause small changes *δ*_m_ and *δ*_f_ in male and female expression relative to the resident allele (Table 2). We assume that the variance of mutational effects *δ*_m_ and *δ*_f_ is fixed to a constant value (Var[*δ*_m_]=Var[*δ*_f_]=0.1). The scope for sexually antagonistic effects is determined by the correlation *ρ* between the effects of mutation in the two sexes, which can be independent (*ρ=*0), similar (*ρ>*0) or opposed (*ρ*<0). The model assumes that gene expression in the homogametic sex is additive and taken to be the sum of the effects of the alleles carried by an individual (Table 2). Expression is assumed to be under stabilizing selection, with the optimum expression arbitrarily set to zero and symmetric decline following a Gaussian function (Table 2). Although stabilizing selection operates in both sexes, its intensity in males and females, denoted by *S*_m_ and *S*_f_, may differ (Table 2).

Assuming that gene expression is at the optimum before degradation of the Y or W allele occurs, the heterogametic sex initially suffers a decrease of *−z*_0_ in expression, where *z*_0_ is the expression contribution of the Y/W chromosome prior to degradation. Expression in the homogametic sex is unaffected and remains at the optimum level. The evolutionary trajectory from this starting point depends on the fixation probability of newly arising mutations. This depends on the sex-specific selection pressure acting on a mutant, given by *S*_m_ and *S*_f_, and the effective population size *N*_eX_ or *N*_eZ_, which scale with the level of sex-specific variance in reproductive success^24^. Iterating this mutation-fixation process and integrating across the distribution of mutational effects *δ*_m_ and *δ*_f_, the expected rates of change in male (*z*_m_) and female (*z*_f_) expression are given by


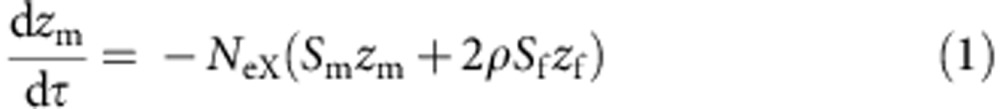



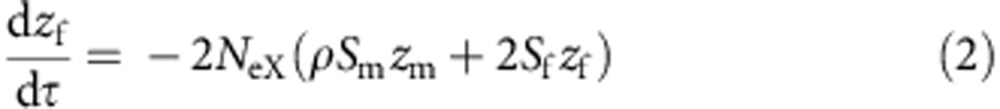



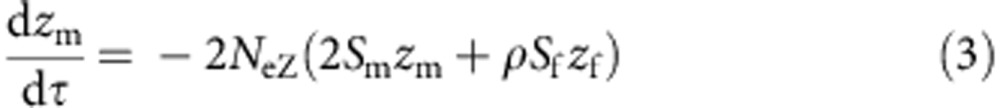



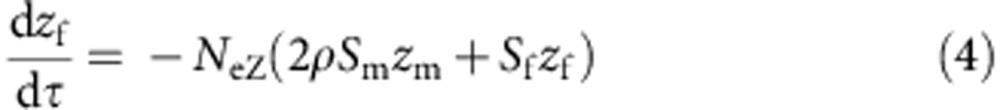


for XY (equations (1) and (2)) and ZW (equations (3) and (4)) species. Evolutionary time *τ* in equations (1)–(4), 2, 3, 4 is scaled with respect to the mutation rate. This means that a unit of *τ* corresponds to fewer generations when the mutation rate is higher. Equations (1)–(4), 2, 3, 4 highlight how the evolution of X- and Z-linked gene expression depends on the asymmetry in sex chromosome dose (two in the homogametic and one in the heterogametic sex), the inter-sexual correlation in expression (*ρ*), the balance between selection in males and females (*S*_m_ and *S*_f_) and the effective population size (*N*_eX_ and *N*_eZ_).

Given sufficient time, the model predicts that both XY and ZW systems will evolve complete dosage compensation for genes whose expression levels depend on gene dose, where expression in the heterogametic sex converges to the ancestral level and males and females reach their respective fitness optima (*z*_m_=0 and *z*_f_=0).

In the absence of any inter-sexual correlation in mutational effects (*ρ=*0), the evolutionary dynamics of dosage compensation are governed by the strength of selection on the heterogametic sex alone. The heterogametic sex returns to the optimal level at an exponential rate 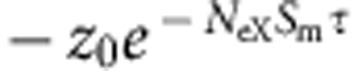 in XY species, and 
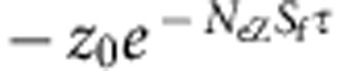
 in ZW species. If the effective population sizes and the mutation rates are the same for the X and Z chromosomes and selection is equivalent in males and females (*S*_m_*=S*_f_), complete dosage compensation will be attained at the same rate in both XY and ZW systems.

**Figure 1:**
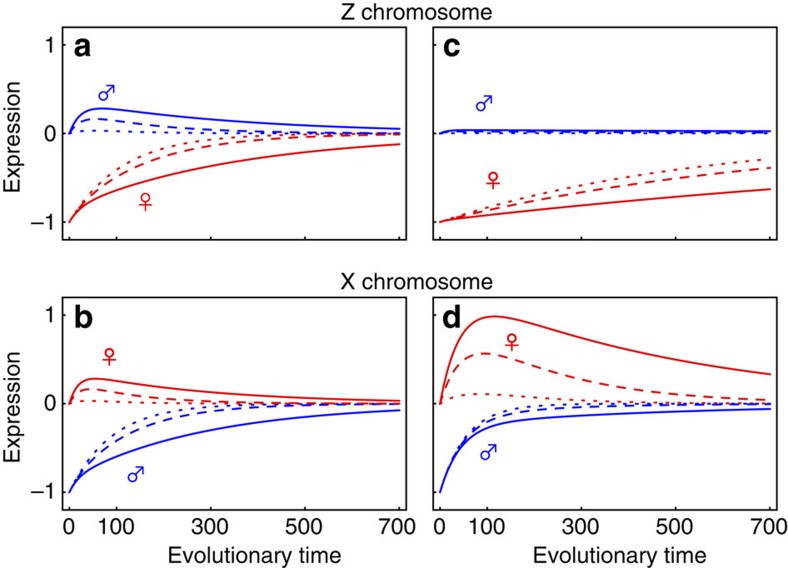
The evolution of dosage compensation on Z and X chromosomes. Expected gene expression is shown for males (blue) and females (red) under a very low (*ρ*=0.1, dotted line), intermediate (*ρ*=0.5, dashed line) and strong inter-sexual correlation (*ρ*=0.8, solid line)—(see equations (13) and (14) for dynamics). **a**,**b** show equal selection in males and females (*S*_m_=*S*_f_=0.5); **c**,**d** show stronger selection in males (*S*_m_=1, *S*_f_=0.1). Evolutionary time refers to the number of generations, ignoring the time taken by successive mutations to fix. Expression is scaled according to the initial degradation *z*_0_ of expression in the heterogametic sex due to the loss of one gene copy, which here is set at *z*_0_=1. Other parameters were also held equal across the two sex chromosome systems (*N*_eX_=*N*_eZ_=1125, *μ*=0.0003).

The speed with which dosage compensation evolves is dramatically reduced under the more plausible assumption that mutations have correlated effects across the sexes (*ρ*≠0). When mutational effects are positively correlated (*ρ>*0), mutations that increase expression and improve dosage compensation in the heterogametic sex are associated with antagonistic fitness effects in the other sex, as they raise expression in the homogametic sex above the fitness optimum. As the homogametic sex carries more chromosomal copies, antagonistic effects impede selection in favour of dosage compensation in the heterogametic sex. If selection is equal between males and females (*S*_m_*=S*_f_), the expectation is that dosage compensation will be slow to evolve both for Z- (Fig. 1a) and X-linked genes (Fig. 1b).

However, selection is typically stronger in males (*S*_m_*>S*_f_) as a result of sexual selection^25^,^26^,^27^,^28^. This creates markedly different evolutionary trajectories of dosage compensation. For Z-linked genes, the combination of male homogamety with more intense selection in males maintains male expression near its optimum (Fig. 1c). Only mutations that increase female expression while minimally affecting males are favoured (Fig. 1c). With a positive inter-sexual correlation for mutational effects (*ρ>*0), the frequency with which such suitable mutations arise is low and the rate at which dosage compensation evolves is very slow (Fig. 1c).

The slow and gradual trajectory of Z-linked genes contrasts with more rapid and sexually antagonistic dynamics for X-linked genes (Fig. 1d). Here more intense selection on males (*S*_m_*>S*_f_) compensates for male X chromosome monosomy, and the evolution of dosage compensation proceeds rapidly. But it does so at a pronounced cost to females, which are displaced from their optimal expression (Fig. 1d). This effect is greater the higher the correlation of mutational effects (*ρ*). Once males approach their optimum expression level, the selective advantage of further change in male expression decreases, and selection on females becomes increasingly important. This causes female expression to slowly evolve back towards the optimum through the fixation of rare mutations that simultaneously relieve the antagonistic effects on female expression while allowing male expression to remain close to its optimum.

Our baseline model assumes additive effects on expression and the instantaneous loss of the W or Y allele. However, we also consider dominance effects on expression and progressive loss of the W or Y allele (see Methods for details). Neither of these variants change our result that dosage compensation evolves faster on the X than on the Z in the presence of more intense selection on males and positive inter-sexual correlation of mutational effects (Supplementary Figs 1 and 2). However, in both cases, the evolutionary trajectory of X expression in females is altered compared with our baseline model. Because selection is more efficient at purging dominant deleterious alleles, the overshoot of X expression in females is mitigated in the presence of dominant mutations (Supplementary Fig. 1). Also, when expression of the Y allele slowly degrades, selection on males to compensate is weaker, and as a result causes weaker detrimental effects to females, whose X expression remains closer to its optimal value (Supplementary Table 2, Supplementary Fig. 2).

### The role of effective population size

**Figure 2:**
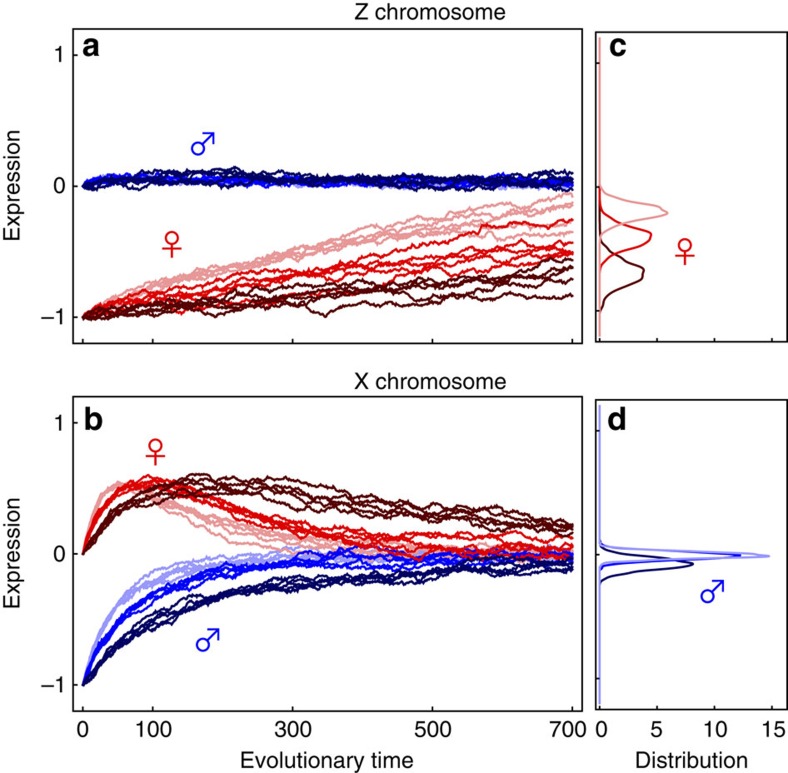
Variance in male reproductive success and the evolution of dosage compensation. Stochastic evolutionary trajectories of male (blue) and female (red) expression for (**a**) Z-linked and (**b**) X-linked genes (see equations (11) and (12) for dynamics). Levels of expression are shown for low (*η=*1, light shade), intermediate (*η=*3, intermediate shade) or high (*η=*10, dark shade) degrees of male reproductive variance (the number of successful breeding females is set at 1,000). A sample of 5 trajectories is shown for each parameter value. Variance in heterogametic sex expression for (**c**) Z-linked and (**d**) X-linked genes after the end of the simulation is shown as a frequency distribution for 1,000 replicates. Selection is stronger on males than on females (*S*_m_=1, *S*_f_=0.2), inter-sexual correlation is relatively strong (*ρ*=0.6) and the mutation rate is *μ*=0.0003.

The evolutionary dynamics at Z- and X-linked genes also depend on the chromosomes' effective population sizes *N*_eZ_ and *N*_eX_ in equations (1)–(4), 2, 3, 4. Larger *N*_*e*_ results in faster evolution of dosage compensation, due to the more reliable fixation of positively selected mutations and elimination of negatively selected mutations (Fig. 2). With effective population size inversely proportional to the stochasticity in replicate evolutionary paths, small values of *N*_e_ not only slow down the evolution of dosage compensation but also result in more variable expression levels across replicate evolutionary trajectories. This effect is observed both during adaptation (Fig. 2), as well as at the evolutionary equilibrium (equation (15)).

Differences in ecology or mating systems (and hence *N*_e_) could underlie variation in the rate at which dosage compensation evolves. Under simplifying assumptions^29^, the effective population sizes of the Z and X chromosomes in a population reproducing under harem polygamy are *N*_eZ_=9*N*_f_/(6+4*η*) and *N*_eX_=9*N*_f_/(6+2*η*), where *N*_f_ is the number of successfully breeding females and *η* is the ratio of the number of successfully breeding females to successfully breeding males. As two-thirds of the Z chromosomes are transmitted by males each generation, compared with only one-third of X chromosomes, greater variance in male reproductive success (*η*>1) leads to a more marked diminution of *N*_eZ_ than *N*_eX_. This predicts slower and more stochastic evolution of dosage compensation on the Z than on the X (Fig. 2).

### Empirical tests of model predictions

The model predicts that whenever selection is more intense on males (*S*_m_*>S*_f_), the evolution of dosage compensation is more rapid and efficient in species with XY as opposed to ZW sex determining systems. This expectation fits broadly with the limited amount of available data on average sex chromosomal expression levels (Table 1). A more rigorous and powerful test can be performed by analysing variation in individual gene expression patterns within sex chromosomes. In particular, an important model prediction is that dosage compensation should be more prevalent among genes subject to strong selection, and in genes where selection is stronger in the heterogametic sex. We addressed these questions using transcriptome data from chicken, a species previously shown to have incomplete Z chromosome dosage compensation^30^. To gauge the strength of selection on individual genes in the two sexes, we measured the sex-specific variability of transcript levels due to differences in expression levels between biological replicates (biological coefficient of variation (BCV); see Methods)^31^,^32^. The assumption is that genes under stronger purifying selection show less expression variation between biological samples. Our approach follows an increasing number of studies using variance as an indicator of selection on gene expression levels^31^,^32^,^33^,^34^,^35^. It is important to note, however, that the assumptions have not been fully validated experimentally, and so should be interpreted with caution.

A set of preliminary analyses supported our estimates of BCV as informative measures for selection strength. First, we observed a negative relationship between BCV and average expression in all tissues and both sexes (Supplementary Table 3). This shows that our estimation procedure successfully removed the positive association between mean and variance that is expected in Poisson-distributed read count data. The negative association is also in line with data from other species^36^ showing that more highly expressed genes are under tighter selective constraint. Second, we also observed a positive association between BCV and the rate of protein evolution (measured as the rate of non-synonymous to synonymous substitutions, *d*_N_/*d*_S_, along the terminal chicken branch; Supplementary Tables 4 and 5). Such an association has been previously observed in vertebrates^37^,^38^,^39^ and insects^33^,^40^,^41^ and implies that genes under more intense purifying selection on expression level also tend to be under stronger purifying selection on protein sequence. Importantly, the positive relationship between expression variance and *d*_N_/*d*_S_ occurs over and above a negative association between average expression level and rates of protein evolution that is observed in other species^42^.

Comparing estimates of male and female BCV, we find that across all autosomal and Z-linked genes expression variation is greater in females than males for all three tissues we analysed (Wilcoxon test on difference between male and female BCV; liver: *V=*851,261, *P*<0.0001, *N=*9,196; heart: *V=*21,797,411, *P*<0.0001, *N=*9,790; gonad: V=1,240,318, *P*<0.0001, *N=*11210). The same difference is also found when analysing autosomal genes alone (liver: *V=*698,999, *P*<0.0001, *N=*8,333; heart: *V=*17,969,183, *P*<0.0001, *N=*8,857; gonad: V=971,978, *P*<0.0001, *N=*10,086). These results support the notion that selection is more intense on male expression.

We analysed whether the degree of dosage compensation of individual Z-linked genes (measured as log_2_ of the ratio of male-to-female expression, see Methods) is related to our measure of selection strength on expression in the two sexes. Because the strength of selection in males and females is positively correlated across loci, we first transformed our measures of male and female expression variability into two perpendicular components, one that measures the strength of sexually concordant selection on expression and the other that measures male (or female) bias in selection intensity. We then related the pattern of dosage compensation across genes expressed in three tissues (heart, liver and gonad) to their value along these axes of selection.

**Figure 3:**
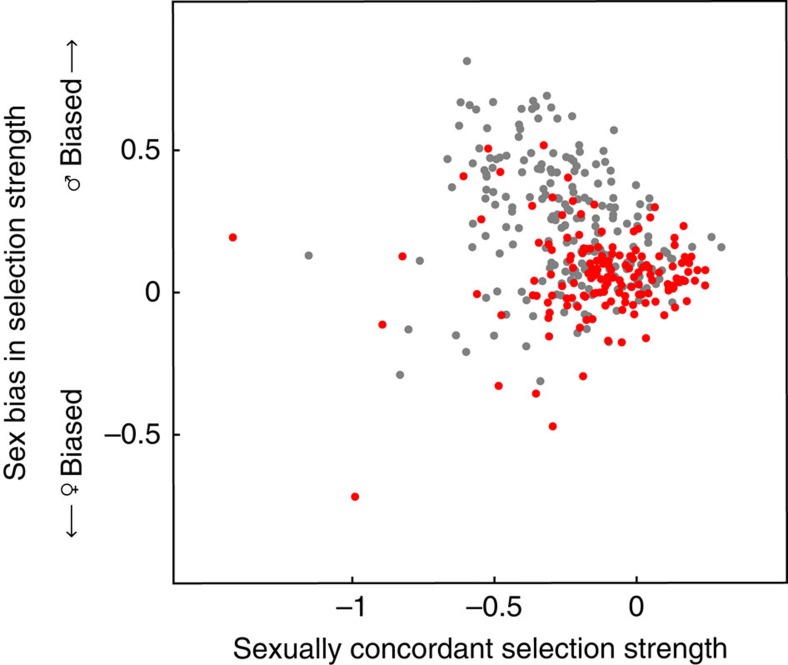
Selection on gene expression and dosage compensation in the chicken liver. The figure shows the strength of sexually concordant selection and the measure of male bias in selection strength for liver-expressed genes used in our analysis. Dosage compensated genes (0.8<male-to-female expression ratio<1.2) are shown in red, and non-dosage compensated genes are shown in grey.

We found that both measures of selection have a significant effect on the male-to-female expression ratio, but that this effect differs between the tissues (tissue-by-selection interactions in Supplementary Table 6). The nature of these differences is best illustrated by analyses run separately on data liver, heart and gonad. In liver, we found that the male-to-female expression ratio decreases with the strength of concordant selection and increases with male-biased selection (Supplementary Table 7, Fig. 3). A significant interaction between the two selection measures further showed that the effect of male-bias in selection intensity is more pronounced when the strength of concordant selection is weak. This means that, as predicted by our model, dosage compensation is most pronounced in genes that are under strong selection in both sexes and those where selection intensity is biased towards females. In contrast to liver, genes expressed in heart showed no relationship between male-to-female expression ratio and either selection measure, while in the gonads we detect only a weak signal for a positive relationship between male-to-female expression ratio and concordant selection strength (Supplementary Table 7, Supplementary Fig. 3).

The model makes the additional prediction that the degree of dosage compensation should increase over time. We assessed this by comparing male-to-female expression ratios across three well-supported strata identified within the chicken Z chromosome. These have been estimated to have diverged from the W chromosome for 75–106 mya (Stratum 1), 45–71 mya (Stratum 2) and 36–68 mya (Stratum 3)^43^. Our model makes the qualitative prediction that if dosage compensation evolves slowly (relative to the age of these strata), then genes on older strata should show more similar (less male biased) expression between the sexes than genes on younger strata. However, our data show no relationship between dosage compensation and the age of the strata (Supplementary Table 8). We obtained qualitatively identical results when we examined the female Z to autosomal expression across strata (Supplementary Table 9); however, the power of these analyses is limited due to the low number of genes in each stratum. This suggests that the evolution of effective dosage compensation occurs within a time range shorter than the age of the youngest chicken stratum (36–68 mya). Furthermore, the persistence of many genes that lack dosage compensation across the strata and the absence of a decrease in their frequency through time suggests either that the genes without dosage compensation have no or little effect on fitness or that persistent sex differences in their expression are accommodated by changes elsewhere in the genome.

## Discussion

Our model predicts that XY and ZW systems can differ substantially in the rate with which dosage compensation evolves and the trajectory taken by male and female expression over the course of evolution. In particular, the evolution of dosage compensation will be slower and more stochastic in ZW than XY systems whenever the inter-sexual correlation in mutational effects is strongly positive and coupled to greater selection in males and/or higher variance in male mating success (Figs 1 and 2).

The conditions that are predicted to lead to different evolutionary trajectories of X- and Z-dosage compensation are biologically plausible. First, more intense selection in males due to sexual selection is a standard expectation supported by much empirical evidence^19^ and this is also supported by our chicken transcriptome data. Importantly, the greater intensity of male selection potentially affects a large number of genes. Thus, genes under sexual selection need not directly code for male reproductive traits, such as ornaments or weaponry. More often than not, male reproductive success depends on general condition^44^, which has a much broader genetic basis, including genes with general effects on viability. In many cases, these genes are also important for female function and therefore under selection for similar expression in both sexes. However, sexual selection tightens the selection pressure for expression in males to be close to the optimum compared with selection on female expression. Second, the situation where sexual selection on males is associated with higher variance in male mating success is frequent in natural populations^18^,^19^. And finally, it is plausible to expect a strong positive correlation of mutational effects on gene expression in males and females. The sexes typically show positive genetic correlations for the expression of genes (for example, refs 13, 14) and new mutations tend to have positively correlated effects on male and female fitness and phenotypes^45^,^46^.

The predictions of our model find some support in our analysis of expression variation on the chicken Z chromosome. Our analyses show that in the liver, the degree of dosage compensation of individual genes is more pronounced in genes that are presumably under strong selection in both sexes, and therefore show reduced expression variance, and where the intensity of selection is greater in females than males (Fig. 3, Supplementary Table 7). Similar relationships were not detected in the other two tissues analysed. In the gonad, expression evolution is likely to be strongly influenced by sex-specific selection pressures that could mask the effects we are interested in. However, the difference between liver and heart is not straightforward to explain. It is conceivable that the function of the liver is more sensitive to changes in gene expression. In line with this argument, liver-expressed genes are on average more dosage compensated than those expressed in the heart (results not shown). More research is needed to elucidate the ultimate causes for tissue differences in regulatory evolution.

Our analysis of gene expression on the chicken Z chromosome also revealed that the prevalence of dosage compensation is independent of the age of evolutionary strata in which the genes reside. This suggests that dosage compensation is saturated within the time-frame of the youngest stratum of the chicken Z chromosome (36–68 mya) and there does not appear to be compensation of additional loci beyond this time. One possible reason for this is that the expression of other Z-linked genes is not or is no longer sensitive to gene copy number; perhaps because other unlinked compensatory changes have already taken place. Alternatively, selection against hypertranscription in the homogametic sex may be sufficiently strong to retard further evolution of compensation in the heterogametic sex. In addition, it is possible that the low effective population size of the Z chromosome limits the efficacy of selection for dosage compensation, and slows its evolution in genes whose expression only mildly depends on dosage, perhaps again favouring the evolution of compensatory mechanisms elsewhere in the genome. However, we note that the youngest stratum in chicken is several tens of millions of years old, so is not young on an absolute scale. Further studies are needed in species with younger sex chromosomes or younger strata to investigate how quickly a plateau is reached in the rate of dosage compensation evolution of Z chromosomes.

The model shows that dosage compensation on the X chromosome evolves via a phase of sexually antagonistic gene expression due to a positive inter-sexual correlation in mutational effects and stronger selection on males. Selection on the heterogametic sex to increase expression towards the optimum is predicted to result in overshooting of the optimum in the homogametic sex. There is some evidence for this in the flour beetle *Tribolium castaneum*, where the female X chromosome is thought to be hypertranscribed relative to autosomal expression levels but the X and autosomes are in dosage parity in males^47^. Sexual conflict over optimal expression may also be the biological explanation for compensatory mechanisms that reduce expression of the X in the homogametic sex. Female-limited mechanisms that re-establish expression parity between the female X chromosomes and autosomes are selected once XY males have approached the optimum expression level (Fig. 1). Female X inactivation in eutherian mammals^48^ and the *Dpy* complex in *C. elegans* hermaphrodites^49^ might be examples of such mechanisms, as could be the repression of X hypertranscription during the embryogenesis of female *Drosophila*^50^.

The model also predicts an important role for reproductive variance and genetic drift in generating differences between species with XY systems and those with ZW systems. In monogamous species, the sex-specific reproductive variance is more often close to equal between males and females (*η*≈1), resulting in similar sex chromosome to autosomal effective population sizes for X and Z chromosomes (*N*_eX_/*N*_eA_≈*N*_eZ_/*N*_eA_≈3/4). Based on our model, we then expect dosage compensation to evolve at similar rates on the X and Z. In polygynous species, however, the greater reproductive skew in males decreases the effective size of the Z much more than that of the X, as there are two copies of the Z in males (*N*_eX_/*N*_eA_>3/4, *N*_eZ_/*N*_eA_<3/4; ref. 51). This asymmetric change in effective population sizes is expected to result in more rapid and less stochastic evolution of dosage compensation on the X compared with the Z (Fig. 2).

Unlike differences in selection that act on individual genes on the X and Z, differences in the mating system, and hence *N*_eX_ and *N*_eZ_, affect the evolution of dosage compensation across the whole X and Z chromosome. They are therefore more apt at explaining differences in average chromosome-wide dosage compensation between species. Our preliminary comparison suggests that dosage compensation is more pronounced in species with XY systems than those with ZW systems (Table 1). Testing the contribution of reproductive variance and genetic drift will require improved comparative analyses, on a larger sample of XY, as well as ZW species that show lineage-specific variation in mating systems. One complication of such analyses will be that the average difference in selection intensity between the sexes is expected to covary with the mating system. Polygynous species are expected to feature increased male reproductive variance, as well as more intense selection on males. As a consequence, disentangling the effects of selection and drift may not be straightforward.

We used a number of assumptions in our modelling to reach tractable results (considered further in the Supplementary Discussion). The model's predictions show a striking similarity to currently described patterns of dosage compensation, and to differences in dosage compensation among genes on the chicken Z. This suggests that the marked difference in dosage compensation between male and female heterogametic species can be explained by the combination of genetic correlation for expression across the sexes, with greater male specific selection and high male reproductive variance. Sexual selection on males simultaneously increases the strength of selection and reproductive variance in males, and thus provides a simple and general explanation for the differences in the evolution of dosage compensation.

## Methods

### Population genetic model of dosage compensation evolution

We model the co-evolution of male and female gene expression level for X- and Z-linked dose-sensitive genes. It is assumed that the Y and W copies have undergone degradation and have no relevant gene expression. In the following, we consider X-linked genes; the Z-linked case is derived in a similar manner.

Gene expression evolves by the recurrent substitutions of mutations that have small sex-specific effects, in a population that in effect remains monomorphic (for example, see refs 52, 53). The population is initially genetically homogeneous, and expression of the X-linked gene is set at *z*_m_ in males and *z*_f_ in females. A *cis*-mutation arises and changes male and female expression by *δ*_m_ and *δ*_f_ respectively (Table 2). Mutant effects on expression are additive, in agreement with data for a large proportion of genes^54^, and appropriate to model dosage compensation, which can be generalized as a *cis*-regulatory additive mechanism^55^,^56^. Mutations are assumed to be sufficiently rare for one mutation to be fixed or lost before another arises^52^. So we can characterize the expected change in expression in the population by considering only the probability of fixation of the mutant, which depends on the life cycle of the population and the fitness effects of the mutation.

The population is composed of *N* males and *N* females, and the life cycle follows a standard Wright–Fisher process^57^. Generations are non-overlapping. Male and female adults produce large numbers of gametes that are randomly paired to produce zygotes. The zygotes are then sampled with replacement and with a selective bias to form the males and females of the next generation. Since the population evolves according to a Wright–Fisher process and selection is weak, we can use Kimura's equation for sex-linked genes to determine the fate of the mutants^57^.

Mutation changes male and female expression level. Whether a mutant is selectively favoured depends on its effects on male and female fitness. It is assumed that there is an optimum expression around which fitness declines. The rate at which fitness decreases from the optimum may differ across the sexes. More precisely, male and female fitness are given as Gaussian functions of the level *z* of expression of the gene, with fitness 
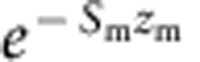
 in males and by 
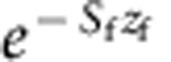
 in females, and the optimum arbitrarily set at zero (*z*_m_=0, *z*_f_=0). Greater values for *S*_m_ and *S*_f_ reflect stronger sex-specific stabilizing selection. It is possible to have different optimal levels in males and females; however, this does not change the qualitative features of our observations.

If the effects of mutations on the level of expression (*δ*_m_, *δ*_f_) are small and the population *N* is large (so that the initial mutation frequency is small), it is reasonable to linearize Kimura's equation for the probability that a single copy X-linked mutant fixes in the population^57^,


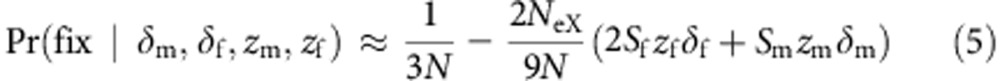


where *N*_eX_ is the effective population size of the X chromosome, and captures the effect of genetic drift, and *N* is the actual population size, capturing the initial frequency of a mutant. The first term of equation (5) is the probability of fixation of a neutral X-linked mutant. The terms inside the bracket are the effects of selection on females and males. Selection increases the probability of fixation if the mutant pushes the expression rate closer to the optimal level (that is, if *z*_f_*δ*_f_<0 or *z*_m_*δ*_m_<0). The increase is proportional to the strength of selection in each sex *S*_f_ and *S*_m_, but is twice as strong in females as they have twice as many X chromosomes compared with males. This lends females a greater importance in the spread of X mutants.

The probability of fixation determines the expected change in expression over one particular substitution in each sex. If the mutant is lost, expression does not change, if the mutant is fixed, male and female expression become *z*_m_+*δ*_m_ and *z*_f_+2*δ*_f_, respectively (Table 2). To calculate the expected change in expression, we consider all possible mutational sizes and their frequencies,


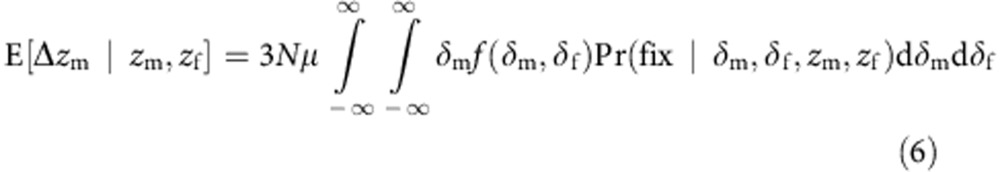



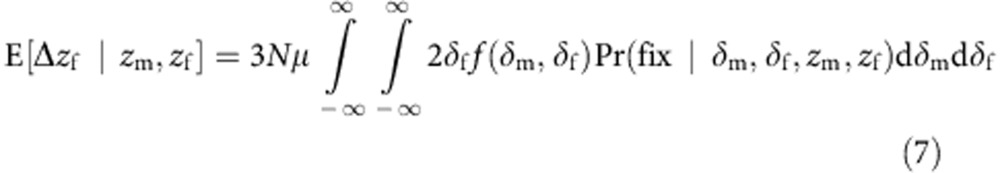


where 3*N* is the total number of genes in the population, *μ* is the mutation rate and *f*(*δ*_m_,*δ*_f_) is the probability density function for the effects of mutants on male and female expression. We assume that *f*(*δ*_m_,*δ*_f_) is a bivariate normal distribution with mean (0, 0) and covariance matrix


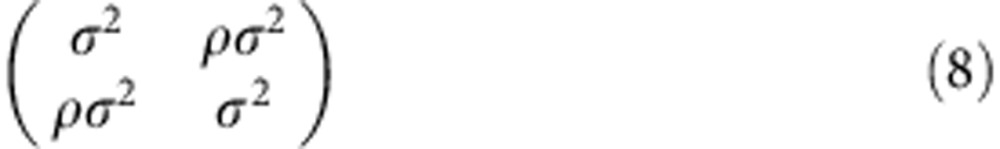


In this case, equations (6) and (7) read


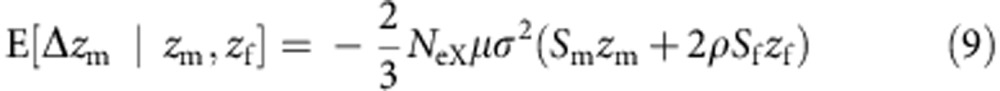



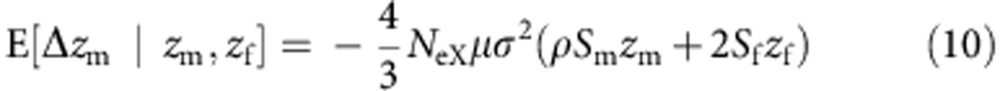


and the second moments of the change in male and female expressions are approximately E[(Δ*z*_m_)^2^|*z*_m_, *z*_f_]=E[(Δ*z*_f_)^2^|*z*_m_*, z*_f_ ]=*σ*^*2*^, and E[(Δ*z*_m_)(Δ*z*_f_ )|*z*_m_*, z*_f_ ]=*ρσ*^2^. For simplicity, we set *σ*^2^=0.1. Setting different values for *σ*^2^ changes the impact of mutations, but since it does so with the same intensity in males and females, it does not change our results. However, increasing *σ*^2^ increases the overall rate of evolution.

Since selection is weak, the first (equations (9) and (10)) and second moments of the distribution of infinitesimal change in male and female expression are sufficient to describe the evolution of male and female expression over many substitutions^52^,^53^. In continuous time, and ignoring the time taken for segregation to occur and the time between mutations, we obtain that the random variables *Z*_m_(*t*) and *Z*_f_(*t*), which describe male and female expression at time *t*, satisfy the stochastic differential equation,


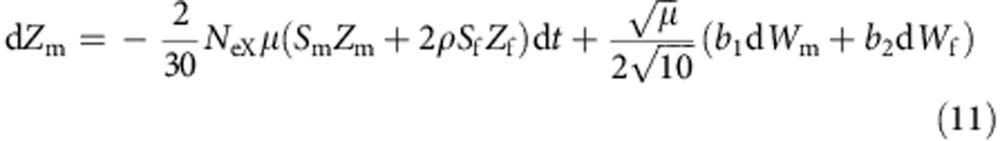



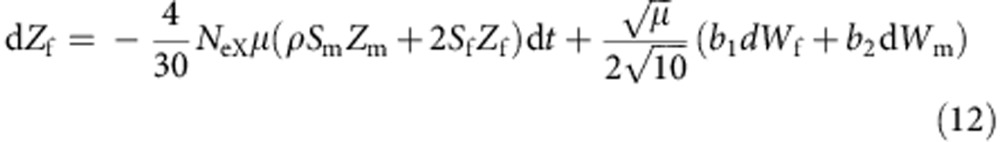


where d*W*_m_ and d*W*_f_ are standard independent Brownian motions, and *b*_*1*_ and *b*_*2*_ are scaled variance terms given by 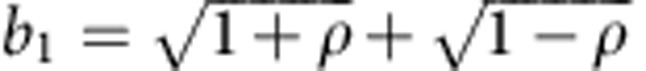 and 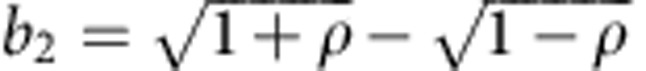.

Unless selection on male and female expression is very weak (very small *N*_eX_*S*_m_, *N*_eX_*S*_f_), and evolution is dominated by genetic drift, the qualitative features of the evolution of dosage compensation are captured by the expected trajectory of equations (11) and (12). Writing the expected male and female expression as *z*_m_ and *z*_f_, we find that their evolution is given by equations (1) and (2), where time is rescaled according to *τ*=2*μt*/30. This scaling eases the comparison between X and Z expression when differences between mutation rates in the two systems are ignored.

The expected values for male and female expression through time, *z*_m_(*τ*) and *z*_f_(*τ*), may be found exactly by solving equations (1) and (2). If we assume that degradation of the Y-linked gene copy leads to an initial diminution in expression in males of −*z*_0_, and female expression is unperturbed by degradation and remains at the ancestral optimal level zero, we have


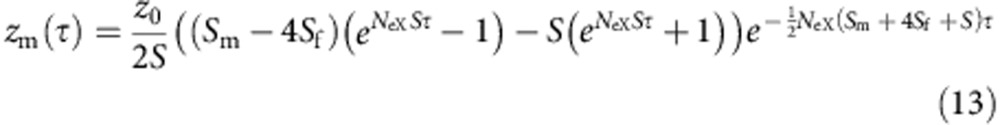



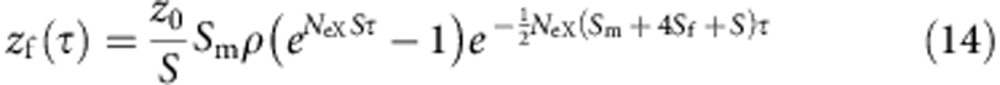


where 
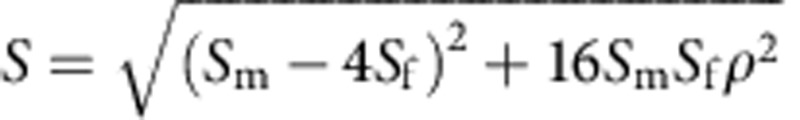
. Similar expressions for Z-linked genes can be found by replacing *N*_eX_ by *N*_eZ_, and m subscripts by f and *vice versa*. Plots in Fig. 1 correspond to equations (13) and (14) with respect to *t* rather than *τ* to compare more easily the deterministic paths with the stochastic replicates of equations (11) and (12) that are shown in Fig. 2.

Using standard results of stochastic systems, the properties of equations (11) and (12) show that after a sufficiently long time, male and female expression will reach an equilibrium distribution that is normally distributed, with mean zero, and a covariance matrix which can be computed explicitly^58^. In particular, the variance in male expression of the X chromosome is given by


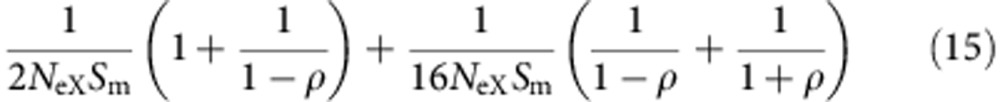


The variance in female expression of the Z chromosome is found by replacing *N*_eX_ by *N*_eZ_ and by substituting *S*_f_ for *S*_m_ in equation (15).

### Effect of dominant mutations

Our model assumes that the expression of X- and Z-linked genes evolve by the accumulation of *cis*-acting mutations, which act locally on the sex chromosomes and therefore are expected to have additive effects on overall expression in the homogametic sex (Table 2). Another non-exclusive possibility is that expression evolves by *trans*-acting mutations. In contrast to *cis* mutants, *trans* mutants are likely to affect the expression of both gene copies in the homogametic sex, and therefore correspond to dominant mutants on overall gene expression.

To illustrate the effect of dominant mutations on the evolution of dosage compensation, we also derive the dynamics of male and female expression when mutants are completely dominant so that expression in heterozygotes is equal to expression in mutant homozygotes of the homogametic sex. In that case, the probability of fixation of a dominant mutant of an X-linked gene is


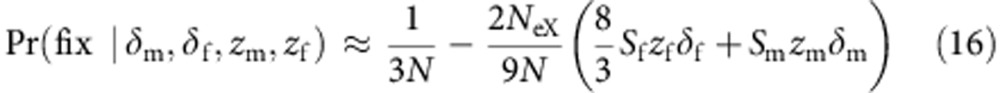


In contrast to the additive case (equation (5)), equation (16) shows that selection on the homogametic females is even stronger. For the evolution of dosage compensation in the presence of correlation in mutational effects across males and females, this implies that counter-selection in the homogametic sex is inflated.

### Effect of continuous expression decrease in the heterogametic sex

Our model investigates the case where the heterogametic sex suffers a decrease in expression due to the loss of expression of the gene copy on the W or Y. An alternative possibility is that once recombination ceases between the sex chromosomes the loss of expression is a result of gradual gene deterioration. To study the effect of a continuous decrease in expression on the evolution of dosage compensation, a time-dependent decline in the expression level of the W- or Y-gene copy is incorporated.

We consider the case where gene expression in the heterogametic sex is initially equal on both sex chromosomes. Starting from half the optimal level (*z*_0_/2), the expression of the W- or Y-gene copy then declines over time *t* according to


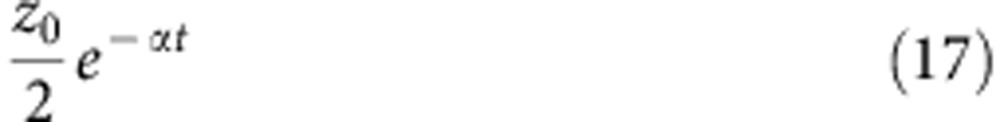


which models an asymptotic decrease approaching total shut-down as time progresses. The parameter *α*>0 controls the rate of decrease in expression of the gene copy. We can then study *cis*-regulatory evolution of the X- or Z-linked copy in males and females in the context of the declining Y- or W-linked gene expression. Fitness in the heterogametic sex then depends on expression from the evolving expression of the Z- or X-gene copy and time-dependent level of expression from the W- or Y-gene copy, while fitness in the homogametic sex depends on expression from both Z- or X-gene copies (Supplementary Table 2).

### Expression analysis

All samples were collected with approval by the Zoology Ethical Review Committee and in accordance with national guidelines. We obtained fertilized eggs of the White Leghorn chicken breed and kept them under standard incubator conditions. The left gonad, heart and liver were collected at embryonic day 19 and stored in RNAlater (Qiagen) until preparation. RNA from each tissue was prepared with the Animal Tissue RNA Kit (Qiagen). Library and RNA-sequence samples were prepared by the Wellcome Trust Centre for Human Genetics Facility at Oxford University using standard methods. Four samples per sex were prepared for each of the four tissues, and each individual was barcoded so that individual variation in coding sequence and expression level could be tracked. Samples were sequenced using Illumina HiSeq as paired-end 100-bp reads, resulting in 16 million paired-end mappable reads per sample, on average. To minimize technical sources of variance, the same HiSeq machine was used for all samples.

Resulting RNA-Seq data were assessed for quality using FastQC v0.10.1 (ref. 59) and filtered using Trimmomatic v0.22 (ref. 60). Filtered reads were mapped to the chicken reference genome (ENSEMBL version 68 WASHUC2) using TopHat v2.05 (refs 61, 62) and Bowtie2 v2.0.0-beta7 (ref. 63), allowing up to two mismatches per alignment. For each tissue separately, aligned reads were sorted and indexed using Samtools v0.1.18 (ref. 64) and raw read counts extracted using Htseq-count v0.5.4p3 (ref. 59). After removing genes for which no read was detected in any sample, raw read counts were normalized across libraries. We used the weighted trimmed mean of *M*-values method in EdgeR v3.2.4 (ref. 65). To limit the impact of incomplete dosage compensation on this step, normalization factors were estimated based on autosomal genes only. Relative transcript abundances were then estimated for all expressed genes as reads per kilobase of exon per million mapped reads (RPKM).

### Statistical analysis of expression data

To filter out noisy gene expression for each tissue, genes were excluded if they were not expressed in all individuals of at least one sex (log_2_(RPKM)<1 for any sample)^32^. For all genes retained, we determined the male-to-female expression ratio as log_2_(average male RPKM/average female RPKM)=log_2_(average male RPKM)−log_2_(average female RPKM). We further estimated the variability of gene expression as an inverse measure of stabilizing selection on expression levels. To quantify expression variability, we used log_2_ of the BCV as estimated by EdgeR. The package implements Bayesian procedures that—based on a negative binomial model—decompose the across-sample variance in read counts for a gene into a part of technical, Poisson-distributed variance and a part of biological variation that stems from true expression differences between samples. The BCV is the square-root of the estimate biological variation divided by the mean expression level of a gene. We estimated BCVs separately for each tissue and sex. The value of the prior, which governs the relative importance of expression variance across all genes and gene-specific expression variance in the estimation of BCVs, was set to 10. Based on preliminary analysis, this value achieved a good balance between capturing gene-specific effects, while minimizing the number of genes with zero estimates of BCV. Using different priors produced qualitatively equivalent results.

### Validation of BCV as a measure for selection strength

We first performed analyses to validate our estimates of BCVs and their interpretation as meaningful measures of selection on expression levels. First, we verified that the estimation of expression variance had successfully removed Poisson-distributed error in read counts by correlating sex- and tissue-specific estimates of BCV with average expression levels across autosomal and Z-linked genes. Next, we verified the relationship between BCV and selective constraint on protein coding sequences, measured as the rate of non-synonymous to synonymous substitutions (*d*_N_/*d*_S_). *Gallus gallus, Meleagris gallopavo, Taeniopygia guttata* and *Anolis carolinensis* complementary DNA sequences were obtained from Ensembl, and orthologues were established using reciprocal blastn^66^. Orthologous sequences were aligned using PRANK^67^ and only included in subsequent analyses if the length of the alignment without gaps was >33 amino acids. *d*_N_/*d*_S_ for each gene for the terminal *G. gallus* branch was estimated using the branch model in the CODEML package in PAML^68^. Orthologues were excluded if d_S_>2 as double hits and mutational saturation can lead to inaccurate divergence estimates^69^. To avoid noise due to genes with poor rate estimates, orthologues were also only included in subsequent analyses if there was at least one non-synonymous or one synonymous substitution, and the total number of substitutions was greater than three on the terminal *G. gallus* branch.

Because estimates of BCV were negatively correlated with expression levels, and high expression levels have been shown to be associated with selective constraint on coding sequence^42^, we performed multiple regression analysis of rates of protein evolution (*d*_N_/*d*_S_) as a function of average expression level (average RPKM across samples) and BCV (all variables log_2_ transformed). The two predictor variables were entered in this specific order to estimate the association between BCV and rates of protein evolution over and above the effect of average expression levels. Analyses were performed separately for each sex with expression levels and BCV averaged across males and females, as well as separately for each tissue and sex.

### Dosage compensation across Z-chromosomal strata

To analyse variation in dosage compensation across the Z chromosome, we filtered genes based on their male-to-female expression ratio. To avoid biasing our analyses by the confounding effects of sex-biased or sex-limited expression, we removed all genes that had strongly sex-biased expression. Specifically, we removed genes with expression ratios >log_2_ (1.25/0.5) and <log_2_ (1/1.25). This filtering removed a limited number of genes from the somatic tissues (422 of 433 genes retained for the heart and 364 of 381 retained for the liver), but more from the gonads (427/534 retained). For all genes passing this filter, we projected male and female expression variability (log_2_ BCV) onto two perpendicular axes, one describing the intensity of sexually concordant selection strength (with values increasing as selection becomes stronger in males and female) and one describing the male bias in selection intensity (values increasing as selection becomes stronger in males and/or weaker in females). The coordinates of the genes on the two axes were then used to determine the effects of selection on male-to-female expression ratios. Transforming selection intensities in this way eliminates problems in subsequent analyses that arise from the correlation between male and female selection and facilitates the interpretation of results.

We analysed variation in dosage compensation across all Z-linked genes and tissues using linear models of male-to-female expression ratio (dependent variable, as log_2_(male RPKM/female RPKM)) as a function of concordant selection intensity, male bias in selection intensity, tissue and their interactions. In line with previous analyses of dosage compensation, our approach assumes statistical independence between genes expressed in the same tissue. We also assume independence between expression measures from the same genes measured in different tissues. However, this assumption does not affect our conclusions; all reported tissue-specific effects of the selection measures are also detected when performing analyses separately on each tissue-specific data set.

We tested whether the age of chromosomal strata had an effect on the level of dosage compensation, over and above that of the two measures of selection strength. We ran an analysis modelling log_2_ male-to-female expression ratio as a function of the two measures of selection strength, tissue, the age of each gene's chromosomal stratum, as well as all the interactions between these terms. Genes that could not be unambiguously assigned to the three major strata were excluded from this analysis. In addition to assessing dosage compensation using the Z-linked log_2_ male-to-female expression ratio, we also calculated the expression ratio of the single Z chromosome and autosomes in females (Z:AA) and males (ZZ:AA) across each stratum.

## Additional information

**Accession codes**: The raw Illumina reads are deposited at the NCBI Sequence Read Archive under Project number: PRJNA284655.

**How to cite this article:** Mullon, C. *et al*. Evolution of dosage compensation under sexual selection differs between X and Z chromosomes. *Nat. Commun.* 6:7720 doi: 10.1038/ncomms8720 (2015).

## Supplementary Material

Supplementary Information Supplementary Figures 1-3, Supplementary Tables 1-9, Supplementary Discussion and Supplementary References

## Figures and Tables

**Table 1:** Current status of dosage compensation.

**Species or clade**	**Karyotype (female:male)**	**Average sex chromosome dosage compensation**
*Caenorhabditis elegans*	XX:XO	Complete
*Drosophila melanogaster*	XX:XY	Complete
*Teleopsis dalmani*	XX:XY	Complete
*Anopheles gambiae*	XX:XY	Complete
Tribolium castaneum*	XX:XY	Complete in males
*Xenos vesparum*	XX:XY	Incomplete in new region, complete in old region shared with *Tribolium*
*Gasterosteus aculeatus*	XX:XY	Incomplete
*Ornithorhynchus anatinus*†	XX:XY	Incomplete
*Monodelphis domestica*	XX:XY	Complete
Eutherian mammals‡	XX:XY	Complete for dosage sensitive genes
*Silene latifolia*	XX:XY	Unclear
*Rumex hastatulus*	XX:XY	Incomplete
*Schistosoma mansoni*	ZW:ZZ	Incomplete
Lepidoptera	ZW:ZZ or ZO:ZZ	Ranges from incomplete to complete
*Cynoglossus semilaevis*	ZW:ZZ	Incomplete
Serpentes	ZW:ZZ	Incomplete
Aves	ZW:ZZ	Incomplete

Recent studies (see Supplementary Table 1 for references) have assessed the presence or absence of complete dosage compensation by comparing the average X or Z expression to the average autosomal expression in the heterogametic (X_male_:AA_male_ or Z_female_:AA_female_) and homogametic (XX_female_:AA_female_ or ZZ_male_:AA_male_) sex.

^*^Tribolium exhibits X chromosome dosage compensation in males, although this appears to have resulted in hyperexpression of X-linked genes in females^47^.

^†^The Platypus has five separate X chromosomes (X_1_, X_2_, X_3_, X_4_, X_5_), which are diploid in females and matched by five Y chromosomes (Y_1_, Y_2_, Y_3_, Y_4_, Y_5_) in males.

^‡^Recent work in the therian mammals indicates that dosage-sensitive genes are compensated in both sexes^48^, through the evolution of female X chromosome inactivation and upregulation of the single active X in both sexes. However, X:AA and XX:AA ratios are <1 for a small number of dosage-insensitive genes and genes with active Y homologues.

**Table 2:** Sex-specific expression in male and female heterogametic systems.

	**Male**			**Female**		
	**Genotype**	**Expression**	**Fitness**	**Genotype**	**Expression**	**Fitness**
Male heterogamety (XY)	R	*z*_m_	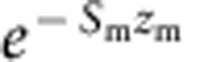	RR	*z*_f_	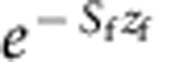
				RM	*z*_f*+*_*δ*_f_	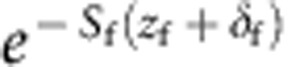
	M	*z*_m_+*δ*_m_	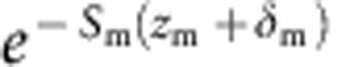	MM	*z*_f*+*_2*δ*_f_	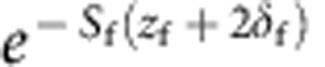
Female heterogamety (ZW)	RR	*z*_m_	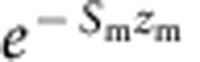	R	*z*_f_	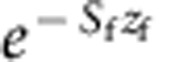
	RM	*z*_m_+*δ*_m_	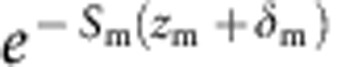			
	MM	*z*_m_+2*δ*_m_	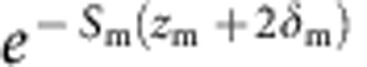	M	*z*_f_+*δ*_f_	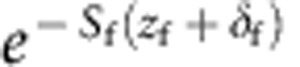

The resident allele (R) has expression level *z*_m_ in males and *z*_f_ in females. The mutant allele (M) causes a small quantitative shift of *δ*_m_ and *δ*_f_ in males and females, respectively.
