# Supplementary material for: Evolution of dosage compensation under sexual selection differs between X and Z chromosomes
Source: Nat Commun. 2015 Jul 27;6:7720. doi: 10.1038/ncomms8720 (PMC4525201; doi:10.1038/ncomms8720)
Supplement: Supplementary Information — Supplementary Figures 1-3, Supplementary Tables 1-9, Supplementary Discussion and Supplementary References [file ncomms8720-s1.pdf]

Supplementary Figure 1

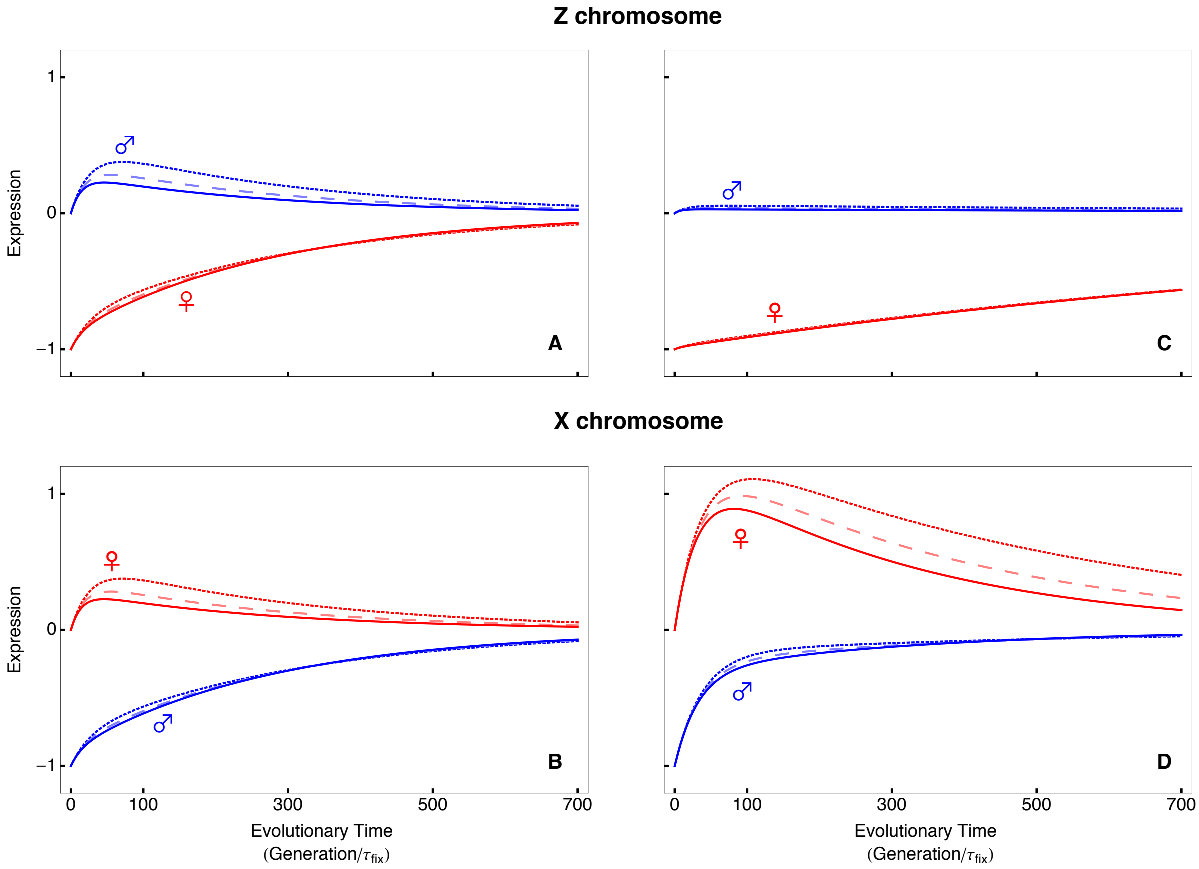

The effect of mutant dominance on the evolution of dosage compensation. Gene expression evolution is shown for males (blue) and females (red) when mutants are recessive (dotted line), additive (dashed line) and dominant (full line). In many cases, the different curves overlap. **A** and **B** show equal selection in males and females ($S_m = S_f = 0.5$); **C** and **D** show stronger selection in males ($S_m = 1$, $S_f = 0.1$). The initial degradation of expression in the heterogametic sex is set at -1. Other parameters were held equal across the two sex chromosome systems in order to aid comparison (i.e., $\rho = 0.8$, $N_{ex} = N_{ez} = 1125$, $\mu = 0.0003$). Dominance has little effect. Rather, the overshoot in the expression of the homogametic sex is mitigated when mutants are dominant. This is because the phenotypic effects on the homogametic sex are initially amplified when mutants are dominant, and as a consequence, selection can pick up mutants that are beneficial to that sex more easily and drive them to fixation. Similarly, because recessive mutants initially cause weaker phenotypic effects on the homogametic sex, the overshoot in expression is amplified when mutations are recessive.

Supplementary Figure 2

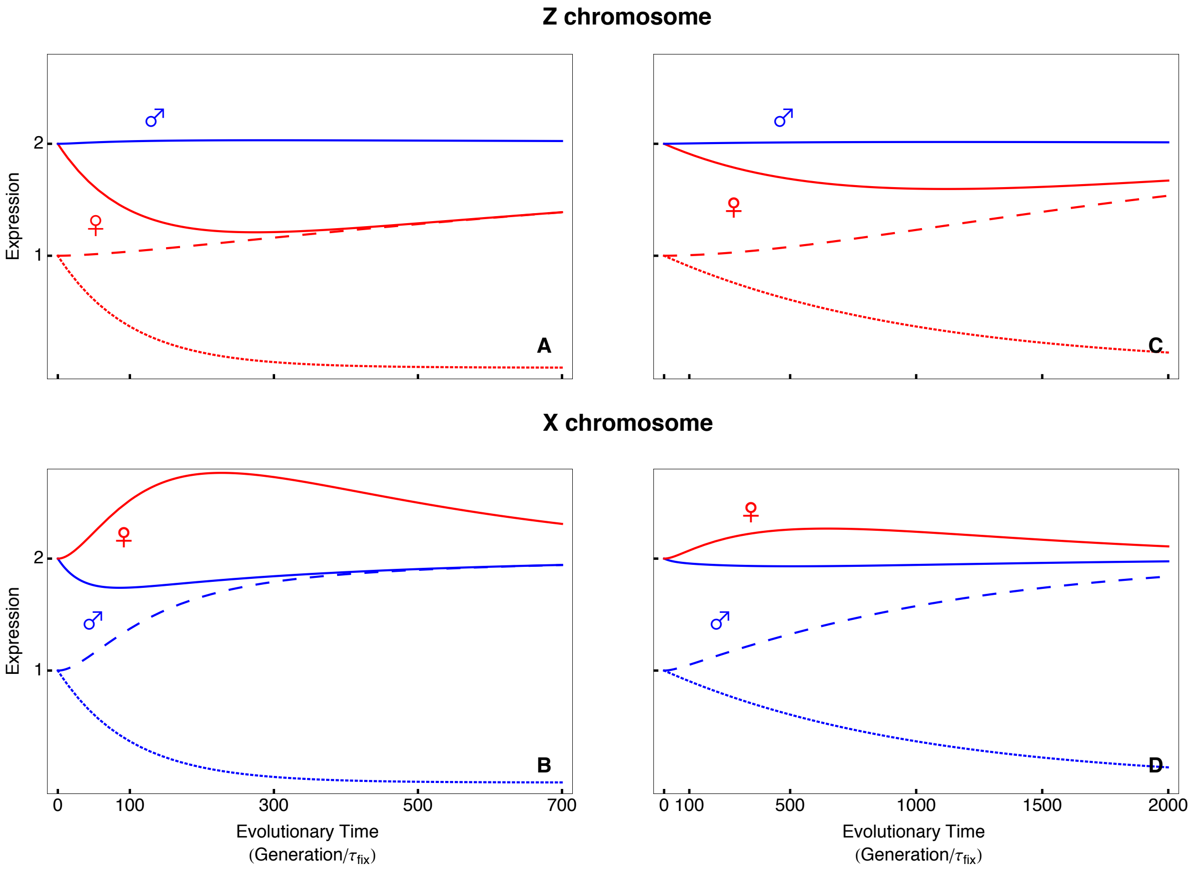

The effect of gradual W- or Y-degradation on the evolution of dosage compensation. Gene expression evolution is shown for males (blue) and females (red), of the W- or Y-copy (dotted line), of the Z- or X-copy in the heterogametic sex (dashed line), and of the total expression of both gene copies (full line). **A** and **B** show rapid decay of the W- or Y-chromosome (α = 0.01); **C** and **D** show slower decay (α = 0.001). The optimal total level of expression in both sexes is set at 2, and selection is stronger on males than on females (S_m = 1, S_f = 0.1). Other parameters were held equal across the two sex chromosome systems (i.e., ρ = 0.8, N_{eX} = N_{eZ} = 1125, μ =0.0003). As the expression from one gene copy is lost in the heterogametic sex, expression of the other copy increases, so that overall expression in the heterogametic sex initially decreases but then converges back to its ancestral level. If selection is stronger on males, female expression is initially offset from its optimal level in XY systems. Unsurprisingly, with a slower decay of expression of the Y-linked gene copy, the evolution of dosage compensation is slower (2C-D). In that case, the offset in female X expression is weaker (2D). This is because selection on the heterogametic sex for compensation is weaker when the decay of the W- or Y-gene copy is slow, and it can therefore evolve with less detrimental effects to the homogametic sex.

Supplementary Figure 3

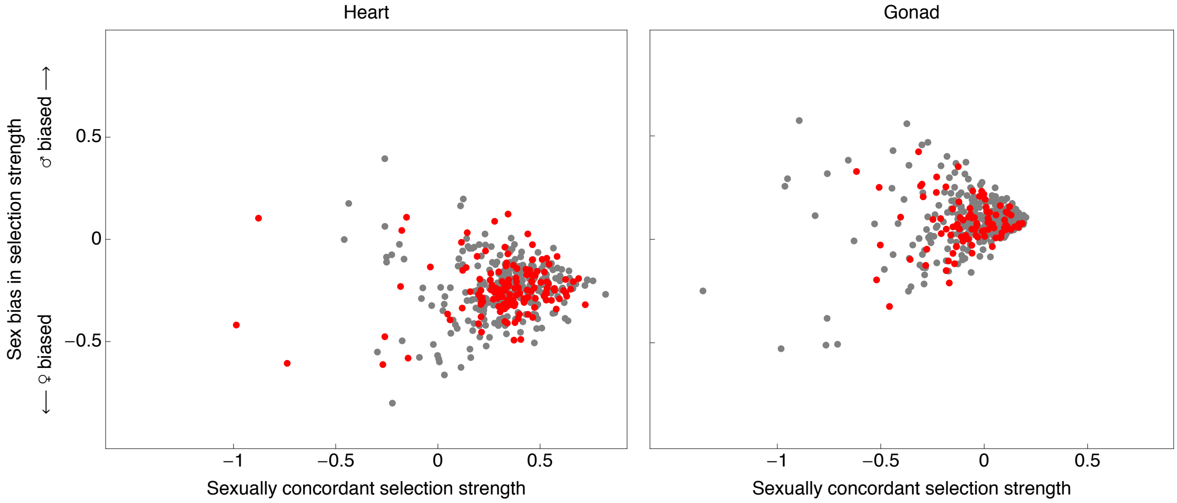

Selection on gene expression and dosage compensation in the chicken heart and gonad. The figure shows the strength of sexually concordant selection and the measure of male bias in selection strength for the heart- and gonad-expressed genes used in our analysis. Dosage compensated genes (0.8 < male-to-female expression ratio < 1.2) are shown in red, and non-dosage compensated genes are shown in grey.

Supplementary Table 1

Species or clade	References
Caenorhabditis elegans	[1, 2, 3]
Drosophila melanogaster	[3, 4]
Teleopsis dalmani	[5]
Anopheles gambiae	[6, 7]
Tribolium castaneum	[8]
Xenos vesparum	[9]
Gasterosteus aculeatus	[10, 11, 12]
Ornithorhynchus anatinus	[13]
Monodelphis domestica	[13]
Eutherian mammals	[13, 14, 15]
Silene latifolia	[16, 17, 18]
Rumex hastatulus	[19]
Schistosoma mansoni	[20]
Lepidoptera	[21, 22, 23, 24]
Cynoglossus semilaevis	[25]
Serpentes	[26]
Aves	[27, 28]

References for current status of dosage compensation.

Supplementary Table 2

	Male			Female		
	**Genotype**	**Expression**	**Fitness**	**Genotype**	**Expression**	**Fitness**
Male heterogamety (XY)	R	$z_\mathrm{m}+\frac{z_0}{2}e^{-\alpha t}$	$e^{-S_\mathrm{m}\left(z_\mathrm{m}+\frac{z_0}{2}e^{-\alpha t}\right)}$	RR	$2z_\mathrm{f}$	$e^{-2S_\mathrm{f}z_\mathrm{f}}$
				RM	$2z_\mathrm{f}+\delta_\mathrm{f}$	$e^{-S_\mathrm{f}(2z_\mathrm{f}+\delta_\mathrm{f})}$
	M	$z_\mathrm{m}+\delta_\mathrm{m}+\frac{z_0}{2}e^{-\alpha t}$	$e^{-S_\mathrm{m}\left(z_\mathrm{m}+\delta_\mathrm{m}+\frac{z_0}{2}e^{-\alpha t}\right)}$	MM	$2(z_\mathrm{f}+\delta_\mathrm{f})$	$e^{-2S_\mathrm{f}(z_\mathrm{f}+\delta_\mathrm{f})}$
Female heterogamety (ZW)	RR	$2z_\mathrm{m}$	$e^{-2S_\mathrm{m}z_\mathrm{m}}$	R	$z_\mathrm{f}+\frac{z_0}{2}e^{-\alpha t}$	$e^{-S_\mathrm{f}\left(z_\mathrm{f}+\frac{z_0}{2}e^{-\alpha t}\right)}$
	RM	$2z_\mathrm{m}+\delta_\mathrm{m}$	$e^{-S_\mathrm{m}(2z_\mathrm{m}+\delta_\mathrm{m})}$			
	MM	$2(z_\mathrm{m}+\delta_\mathrm{m})$	$e^{-2S_\mathrm{m}(z_\mathrm{m}+\delta_\mathrm{m})}$	M	$z_\mathrm{f}+\delta_\mathrm{f}+\frac{z_0}{2}e^{-\alpha t}$	$e^{-S_\mathrm{f}\left(z_\mathrm{f}+\delta_\mathrm{f}+\frac{z_0}{2}e^{-\alpha t}\right)}$

Sex-specific expression in male and female heterogametic systems with gradual expression decrease in heterogametic sex. See methods for details. The resident allele (R) causes expression level z_m in males and z_f in females of the Z- or X-gene copy. The mutant allele (M) causes a small quantitative shift of δ_m and δ_f in males and females respectively. Meanwhile, the expression of the W- or Y-gene copy depends on time t, and is independent from mutations affecting expression of the Z- or X-gene copy.

Supplementary Table 3

Tissue	N	Sex	Spearman's rho	P-value
Liver	9196	Male	-0.229	<0.001
		Female	-0.234	<0.001
Heart	9790	Male	-0.267	<0.001
		Female	-0.251	<0.001
Gonad	11210	Male	-0.418	<0.001
		Female	-0.305	<0.001

Correlations between BCV and average expression levels. The table shows the results of correlation tests between BCV and average expression (RPKM). Tests were performed across autosomal and Z-linked genes and performed separately for each tissue and sex. The table provides the sample size (N), the estimate of the Spearman's rank correlation coefficient rho and the associated P-value, based on an asymptotic *t* approximation.

Supplementary Table 4

Tissue	Predictor	F	P_F	b	P_t
Liver	Expression level	4.319	0.039	-0.029	0.096
	BCV	34.132	<0.001	0.677	<0.001
Heart	Expression level	57.112	<0.001	-0.101	<0.001
	BCV	14.002	<0.001	0.424	<0.001
Gonad	Expression level	125.327	<0.001	-0.175	<0.001
	BCV	6.518	0.011	0.257	0.011

Tissue-specific relationships between rates of protein evolution and average expression levels and BCV in males and females. The table shows the results of multiple regression of d_N/d_S on expression levels and BCV averaged across males and females. Analyses were performed on log_2-transformed data and separately for each tissue. For each predictor, the table provides the F ratio and associated P-value from an Analysis of Variance performed on the regression model, as well as the estimated slope and it's associated P-value from a t-test comparing the estimate to zero. We therefore find a significant association between BCV and rates of protein evolution. Thus, BCV has a positive significant effect on $d_N/d_{S,}$ even when the (negative) effect of average expression level has been accounted for.

Supplementary Table 5

a) Males

Tissue	Predictor	F	P_F	b	P_t
Liver	Expression level	4.448	0.035	-0.023	0.092
	BCV	31.929	<0.001	0.543	<0.001
Heart	Expression level	59.124	<0.001	-0.108	<0.001
	BCV	3.231	0.072	0.176	0.072
Gonad	Expression level	111.903	<0.001	-0.157	<0.001
	BCV	2.847	0.092	0.092	0.092

b) Females

Tissue	Predictor	F	P_F	b	P_t
Liver	Expression level	4.333	0.037	-0.025	0.068
	BCV	19.334	<0.001	0.472	<0.001
Heart	Expression level	55.105	<0.001	-0.097	<0.001
	BCV	22.984	<0.001	0.484	<0.001
Gonad	Expression level	1299.661	<0.001	-0.179	<0.001
	BCV	6.989	0.008	0.250	0.008

Tissue and sex-specific relationships between rates of protein evolution and expression levels and BCV. The table shows the results of multiple regression of d_N/d_S on expression levels and BCV. Analyses were performed on log_2-transformed data and separately for each sex and tissue. For each predictor, the table provides the F ratio and associated P-value from an Analysis of Variance performed on the regression model, as well as the estimated slope and it's associated P-value from a t-test comparing the estimate to zero. Some of the significance tests lack power due to the collinearity between average expression and BCV (see Supplementary Table 2). However, in all of these cases the BCV term is significant when entered first, ahead of expression level (results not shown).

Supplementary Table 6

Model term	df	SS	MS	F	P-value
Sel_{conc}	1	0.107	0.107	0.988	0.32
$Sel_{M\text{-}to\text{-}F}$	1	1.818	1.818	16.736	<0.0001
Tissue	2	17.140	8.570	78.881	<0.0001
Sel_{conc}: $Sel_{M\text{-}to\text{-}F}$	1	0.023	0.023	0.210	0.65
Sel_{conc}:Tissue	2	1.816	0.908	8.356	0.0003
$Sel_{M\text{-}to\text{-}F}$:Tissue	2	1.099	0.550	5.059	0.0065
Sel_{conc}: $Sel_{M\text{-}to\text{-}F}$:Tissue	2	0.059	0.030	0.273	0.76
Residuals	1201	130.483	0.109		

Analysis of Variance of gene expression. The table shows the ANOVA table for a linear model of $\log_2$ male-to-female expression ratio as a function of the strength of sexually concordant selection (Sel_{conc}), male bias in the strength of selection ($Sel_{M\text{-}to\text{-}F}$), tissue and their interactions (indicated by colons). Provided are figures for the degrees of freedom (df), Sums of Squares (SS), Mean Squares (MS), the value of the F-ratio (F) and the associated P-value for each model term.

Supplementary Table 7

Tissue	Model term	df	SS	MS	F	P-value	Slope	P-value
Liver	Sel_{conc}	1	1.816	1.816	35.038	<0.0001	-0.271	<0.0001
	$Sel_{M\text{-to-}F}$	1	2.705	2.705	52.209	<0.0001	0.636	<0.0001
	Sel_{conc} :$Sel_{M\text{-to-}F}$	1	0.303	0.303	5.842	0.0162	0.587	0.0165
	Residuals	360	18.654	0.052				
Heart	Sel_{conc}	1	0.011	0.011	0.112	0.73	0.113	0.29
	$Sel_{M\text{-to-}F}$	1	0.036	0.036	0.367	0.55	0.028	0.81
	Sel_{conc} :$Sel_{M\text{-to-}F}$	1	0.128	0.128	1.290	0.26	0.402	0.26
	Residuals	418	41.542	0.099				
Gonad	Sel_{conc}	1	0.700	0.700	4.212	0.0408	0.149	0.14
	$Sel_{M\text{-to-}F}$	1	0.421	0.421	2.535	0.11	0.371	0.10
	Sel_{conc} :$Sel_{M\text{-to-}F}$	1	0.107	0.107	0.644	0.42	0.263	0.42
	Residuals	423	70.286	0.166				

Tissue-specific linear model of gene expression. The table combines Analyses of Variance and parameter estimates (regression slope and associated P values of a *t*-test against 0) for the effects of sexually concordant and male-biased selection (Sel_{conc} and $Sel_{M\text{-to-}F}$) on $\log_2$ male-to-female expression ratio in liver, heart and gonad.

Supplementary Table 8

**Model term**	**df**	**SS**	**MS**	**F**	**P-value**
Sel_{conc}	1	0.190	0.190	1.750	0.19
$Sel_{M\text{-}to\text{-}F}$	1	0.643	0.643	5.915	0.0152
Tissue	2	8.333	4.167	38.35	<0.0001
Age	1	0.065	0.065	0.600	0.44
Sel_{conc}: $Sel_{M\text{-}to\text{-}F}$	1	0.014	0.014	0.126	0.72
Sel_{conc}:Tissue	2	1.707	0.853	7.853	0.0004
$Sel_{M\text{-}to\text{-}F}$:Tissue	2	0.681	0.341	3.135	0.0441
Sel_{conc}: Age	1	0.077	0.077	0.712	0.40
$Sel_{M\text{-}to\text{-}F}$:Age	1	0.037	0.037	0.338	0.56
Tissue:Age	2	0.053	0.027	0.244	0.78
Sel_{conc}:$Sel_{M\text{-}to\text{-}F}$:Tissue	2	0.005	0.003	0.025	0.98
Sel_{conc}:Tissue:Age	1	0.243	0.243	2.236	0.14
$Sel_{M\text{-}to\text{-}F}$:Tissue:Age	2	0.392	0.196	1.802	0.17
Sel_{conc}:$Sel_{M\text{-}to\text{-}F}$:Tissue:Age	2	0.120	0.060	0.553	0.58
Residuals	750	81.488	0.109		

Linear model of gene expression including stratum age. The table shows the ANOVA table for a linear model of log_2 male-to-female expression ratio as a function of the strength of sexually concordant selection (Sel_{conc}), male bias in the strength of selection ($Sel_{M\text{-}to\text{-}F}$), tissue, stratum age (Age) and their interactions (indicated by colons). Provided are figures for the degrees of freedom (df), Sums of Squares (SS), Mean Squares (MS), the value of the F-ratio (F) and the associated P-value for each model term. Thus, we do not detect an effect of stratum age, nor a significant interaction between stratum age and any of the other variables. Qualitatively identical results were found when modelling stratum number (1-3) instead of stratum age.

Supplementary Table 9

Tissue	Stratum 1		Stratum 2		Stratum 3		Spearman's rho	P-value
	N	Z:AA P-value	N	Z:AA P-value	N	Z:AA P-value		
Liver	20	0.285 p=0.056	33	0.300 p=0.074	190	0.880 p<0.001	-0.739	0.471
Heart	26	0.267 p<0.001	34	0.201 p=0.002	217	0.518 p<0.001	-0.559	0.623
Gonad	35	0.560 p<0.001	39	0.373 p<0.023	263	0.651 p<0.001	-0.075	0.952

Dosage compensation status across Z chromosome strata. The table shows the expression ratio between the single Z chromosome and autosomes in females (Z:AA) in each tissue. Significant differences between Z-linked and autosomal female expression were assessed using Wilcox tests. The Spearman's rank correlation coefficient rho and associated P-value describe the correlation between Z:AA ratio and stratum age. So, Z-linked expression in females is significantly lower than autosomal expression across all strata, with the exception of Strata 1 and 2 in the liver, which are marginally non-significant. This is consistent with a lack of global dosage compensation. However, for each tissue, we find no significant difference in female expression between strata (all p > 0.100) and Spearman's rank correlations reveale no significant correlation between Z:AA ratio and stratum age. There is also no significant difference in male expression between strata (all p > 0.08).

Supplementary Discussion

Care needs to be taken when using the model to generate quantitative predictions, as a number of simplifying assumptions have been made in order to reach tractable results. First, the parameter we use to describe evolutionary time is not the same as real time or numbers of generations. Evolutionary time here ignores periods during which alleles segregate. This should not make any difference to our general conclusions as effective population size has opposing effects on the expected time taken for initially rare adaptive mutants to fix. On the one hand, the probability of fixation of rare adaptive mutations increases with N_e. On the other hand, the number of generations taken by an initially rare mutant to fix increases linearly with N_e [29]. As a result, adaptive rate only increases weakly with effective population size [29]. Strong selection on both sexes could also affect segregation times by delaying the fixation of alleles with sexually antagonistic effects, as opposing forces of selection maintain polymorphism for extended periods of time. However, this concerns only a small subset of possible mutations whose fitness effects in males and females closely balance each other out [30]. Therefore, unless differences in N_e are very large, or selection is very strong, our measure of evolutionary time is a good proxy to compare the evolution of X and Z dosage compensation.

A second simplifying assumption is that we modelled the evolution of expression of a single gene by *cis*-acting mutations with additive effects. Mechanistic models of dosage compensation systems suggest rather a combination of *cis* and *trans* effects that affect expression at a regional level [31]. Mutation in the regulation of *trans* elements should have dominant rather than additive effects on expression in the homogametic sex as *trans* elements will affect the expression of both gene copies. It seems unlikely that this difference will have a large consequence for our model results as we found that dominant, recessive or additive *cis* mutants had little effect on the overall rate at which dosage compensation evolves, but a full investigation of *trans* mutants is needed to confirm this. Third, another consequence of regional regulation is that the expression of multiple genes will be affected by mutation. The conclusions of our model can be readily extended to multiple loci if their expression is under similar selection pressure and their expression has additive effects on fitness. However, it is more complicated to infer the consequences of regional regulation when individual genes are subject to variation in the strength of sex-specific selection and different degrees of imbalance in expression. We nonetheless expect the broad-brush conclusion of our model to hold, and that adaptation on the X to male function should be faster than adaption on the Z to female function.

Finally, variation in the overall mutation rates and sex-differences in mutation rates, need to be considered with care. Male bias in mutation rate is relatively common [32] and would have two consequences. First, it would supply the Z with a greater input of mutants (relative to the X), thereby permitting dosage compensation to evolve faster. Second, it would entail a slower decay of the W relative to the Y [33], which might allow the Z chromosome more time to adapt to dose effects [34], and, as we have shown in our model (Supplementary Figure 2), to evolve with fewer sexually antagonistic effects for the homogametic sex than the X. Our model and the data [35, 36] suggest that selection is generally less effective on Z than X chromosomes, and this will in general counteract any effects resulting from sex difference in mutation rate. Data on dosage compensation from a greater number of species would make it possible to disentangle these effects.

Supplementary References

1. Ercan S, Giresi PG, Whittle CM, Zhang XM, Green RD, Lieb JD. X chromosome repression by localization of the *C elegans* dosage compensation machinery to sites of transcription initiation. *Nature Genetics* **39**, 403-408 (2007).

2. Reinke V, Gil IS, Ward S, Kazmer K. Genome-wide germline-enriched and sex-biased expression profiles in *Caenorhabditis elegans*. *Development* **131**, 311-323 (2004).

3. Straub T, Becker PB. Dosage compensation: the beginning and end of generalization. *Nature Reviews Genetics* **8**, 47-57 (2007).

4. Gelbart ME, Kuroda MI. Drosophila dosage compensation: a complex voyage to the X chromosome. *Development* **136**, 1399-1410 (2009).

5. Wilkinson GS, Johns PM, Metheny JD, Baker RH. Sex-Biased Gene Expression during Head Development in a Sexually Dimorphic Stalk-Eyed Fly. *PLoS ONE* **8**, e59826 (2013).

6. Baker DA, Nolan T, Fischer B, Pinder A, Crisanti A, Russell S. A comprehensive gene expression atlas of sex- and tissue-specificity in the malaria vector, *Anopheles gambiae*. *Bmc Genomics* **12**, (1) (2011).

7. Hahn MW, Lanzaro GC. Female-biased gene expression in the malaria mosquito *Anopheles gambiae*. *Current Biology* **15**, R192-R193 (2005).

8. Prince EG, Kirkland D, Demuth JP. Hyperexpression of the X chromosome in both sexes results in extensive female bias of X-linked genes in the Flour Beetle. *Genome Biology and Evolution* **2**, 336-346 (2010).

9. Mahajan S, Bachtrog D. Partial Dosage Compensation in Strepsiptera, a Sister Group of Beetles. *Genome Biology and Evolution* **7**, 591-600 (2015).

10. Leder EH*, et al.* Female-biased expression on the X chromosome as a key step in sex chromosome evolution in threespine sticklebacks. *Molecular Biology and Evolution* **27**, 1495-1503 (2010).

11. White MA, Kitano J, Peichel CL. Purifying selection maintains dosage-sensitive genes during degeneration of the threespine stickleback Y chromosome. *Molecular Biology and Evolution*, msv078 (2015).

12. Schultheiß R, Viitaniemi HM, Leder EH. Spatial dynamics of evolving dosage compensation in a young sex chromosome system. *Genome Biology and Evolution* **23**, 581-590 (2015).

13. Julien P*, et al.* Mechanisms and Evolutionary Patterns of Mammalian and Avian Dosage Compensation. *PLoS Biology* **10**, e1001328 (2012).

14. Lin FQ, Xing K, Zhang JZ, He XL. Expression reduction in mammalian X chromosome evolution refutes Ohno's hypothesis of dosage compensation. *Proceedings of the National Academy of Sciences, USA* **109**, 11752-11757 (2012).

15. Pessia E, Makino T, Bailly-Bechet M, McLysaght A, Marais GAB. Mammalian X chromosome inactivation evolved as a dosage-compensation mechanism for dosage-sensitive genes on the X chromosome. *Proceedings of the National Academy of Sciences, USA* **109**, 5346-5351 (2012).

16. Muyle A, Zemp N, Deschamps C, Mousset S, Widmer A, Marais GAB. Rapid de novo evolution of X chromosome dosage compensation in *Silene latifolia*, a plant with young sex chromosomes. *PLoS Biology* **10**, e1001308 (2012).

17. Chibalina Margarita V, Filatov Dmitry A. Plant Y Chromosome Degeneration Is Retarded by Haploid Purifying Selection. *Current Biology* **21**, 1475-1479 (2011).

18. Bergero R, Qiu S, Charlesworth D. Gene Loss from a Plant Sex Chromosome System. *Current Biology* **25**, 1234-1240 (2015).

19. Hough J, Hollister JD, Wang W, Barrett SCH, Wright SI. Genetic degeneration of old and young Y chromosomes in the flowering plant Rumex hastatulus. *Proceedings of the National Academy of Sciences* **111**, 7713-7718 (2014).

20. Vicoso B, Bachtrog D. Lack of global dosage compensation in *Schistosoma mansoni*, a female-heterogametic parasite. *Genome Biology and Evolution* **3**, 230-235 (2011).

21. Arunkumar KP, Mita K, Nagaraju J. The silkworm Z chromosome Is enriched in testis-specific genes. *Genetics* **182**, 493-501 (2009).

22. Harrison P, Mank JE, Wedell. Incomplete sex chromosome dosage compensation in the Indian meal moth, Plodia interpunctella, based on de novo transcriptome assembly. *Genome Biology and Evolution* **4**, 1118-26 (2012).

23. Zha X, Xia Q, Duan J, Wang C, He N, Xiang Z. Dosage analysis of Z chromosome genes using microarray in silkworm, *Bombyx mori*. *Insect Biochemistry and Molecular Biology* **35**, 315-321 (2009).

24. Smith G, Chen Y-R, Blissard GW, Briscoe AD. Complete dosage compensation and sex-biased gene expression in the moth Manduca sexta. *Genome Biology and Evolution*, (2014).

25. Chen S, *et al.* Whole-genome sequence of a flatfish provides insights into ZW sex chromosome evolution and adaptation to a benthic lifestyle. *Nat Genet* **46**, 253-260 (2014).

26. Vicoso B, Emerson JJ, Zektser Y, Mahajan S, Bachtrog D. Comparative Sex Chromosome Genomics in Snakes: Differentiation, Evolutionary Strata, and Lack of Global Dosage Compensation. *PLoS Biol* **11**, e1001643 (2013).

27. Arnold AP, Itoh Y, Melamed E. A Bird's-Eye View of Sex Chromosome Dosage Compensation. *Annual Review of Genomics and Human Genetics* **9**, 109-127 (2008).

28. Mank JE. The W, X, Y and Z of sex chromosome dosage compensation. *Trends in Genetics* **25**, 226-233 (2009).

29. Ewens WJ. *Mathematical Population Genetics 1: I. Theoretical Introduction*. Springer (2004).

30. Mullon C, Pomiankowski A, Reuter M. The effects of selection and genetic drift on the genomic distribution of sexually antagonistic alleles. *Evolution* **66**, 3743-3753 (2012).

31. Ercan S. Mechanisms of X Chromosome Dosage Compensation. *Journal of Genomics* **3**, 1-19 (2015).

32. Wilson Sayres MA, Makova KD. Genome analyses substantiate male mutation bias in many species. *BioEssays* **33**, 938-945 (2011).

33. Bachtrog D, *et al.* Are all sex chromosomes created equal? *Trends in Genetics* **27**, 350-357 (2011).

34. Naurin S, Hansson B, Bensch S, Hassequist D. Why does dosage compensation differ between XY and ZW taxa? *Trends in Genetics* **26**, 15-20 (2010).

35. Mank JE, Nam K, Brunström B, Ellegren H. Ontogenetic complexity of sexual dimorphism and sex-specific selection. *Molecular Biology and Evolution* **27**, 1570-1578. (2010).

36. Mank JE, Vicoso B, Berlin S, Charlesworth B. Effective population size and the Faster-X Effect: Empirical results and their interpretation. *Evolution* **64**, 663-674 (2010).
